# Long-Term Recurrence-Free Survival of an Elderly Patient With Diffuse Large B-cell Lymphoma in the Setting of Rituximab-Containing Therapy and a Natural History of Gastric Adenocarcinoma

**DOI:** 10.7759/cureus.68217

**Published:** 2024-08-30

**Authors:** Toshiyuki Kubo, Yasushi Adachi, Satoshi Yamamoto, Toshiya Sakai, Akira Goto

**Affiliations:** 1 Department of Internal Medicine, Sapporo Shirakaba-dai Hospital, Sapporo, JPN; 2 Department of Hematology, Sapporo City General Hospital, Sapporo, JPN; 3 Department of Hematology, National Hospital Organization Hokkaido Cancer Center, Sapporo, JPN

**Keywords:** rituximab, natural history, gastric cancer, diffuse large b-cell lymphoma (dlbcl), burkitt’s lymphoma

## Abstract

Diffuse large B-cell lymphoma (DLBCL) is a high-grade malignancy. We present a case of a 97-year-old female with gastric cancer and DLBCL in whom remission with rituximab-containing minimum chemotherapy was sustained for 10 years. As she had severe adverse effects, she refused further treatments for both tumors. Ten years after the initial treatment, examinations showed several tumors in the lungs, the right pleura, and the liver, as well as advanced gastric cancer. She eventually passed away, and the autopsy revealed that multiple tumors were not lymphoma, but adenocarcinoma. This case report is a valuable addition to the literature as it analyzes whether rituximab-containing minimum chemotherapy is effective for elderly DLBCL and delineates the natural history of gastric cancer.

## Introduction

The classification of malignant lymphomas has been a matter of controversy in the past two decades and has shifted from morphological- to more molecular-based criteria [1,2]. Diffuse large B-cell lymphoma (DLBCL) is the most common variant, accounting for 30-40% of all lymphomas, and is an intermediate- to high-grade malignancy [3]. On the other hand, Burkitt's lymphoma is considered to have a poor prognosis among hematologic diseases [4]. This pathology therefore warrants a more aggressive regimen than the standard chemotherapy regimen for DLBCL, which is currently rituximab, cyclophosphamide, doxorubicin, vincristine, prednisolone (R-CHOP) [4-6]. However, R-CHOP is intolerable for elderly patients and those with severe comorbidities. Rituximab monotherapy is the standard of care for follicular lymphoma, but it has been shown to be ineffective for Burkitt's lymphoma [7].

We report a case that was previously highly suspected to be Burkitt's lymphoma combined with early-stage gastric cancer, with the former remaining in remission for 10 years after initial treatment. However, Burkitt’s lymphoma was diagnosed according to the previous classification and the final diagnosis was changed to DLBCL, not otherwise specified (DLBCL, NOS) according to the present classification [1,2]. Although the diagnostic criteria have changed, this case is still regarded as lymphoma with high-grade malignancy that responded well to rituximab-containing minimum chemotherapy. The patient eventually passed away, and her autopsy revealed only gastric adenocarcinomas, with no recurrences of lymphoma, suggesting the potential effectiveness of rituximab-containing minimum chemotherapy for DLBCL, NOS in the elderly. In addition, as there are few published reports on the natural history of gastric adenocarcinoma, this case report provides important observations on the natural history of gastric cancer over a 10-year period.

## Case presentation

**Figure 1:**
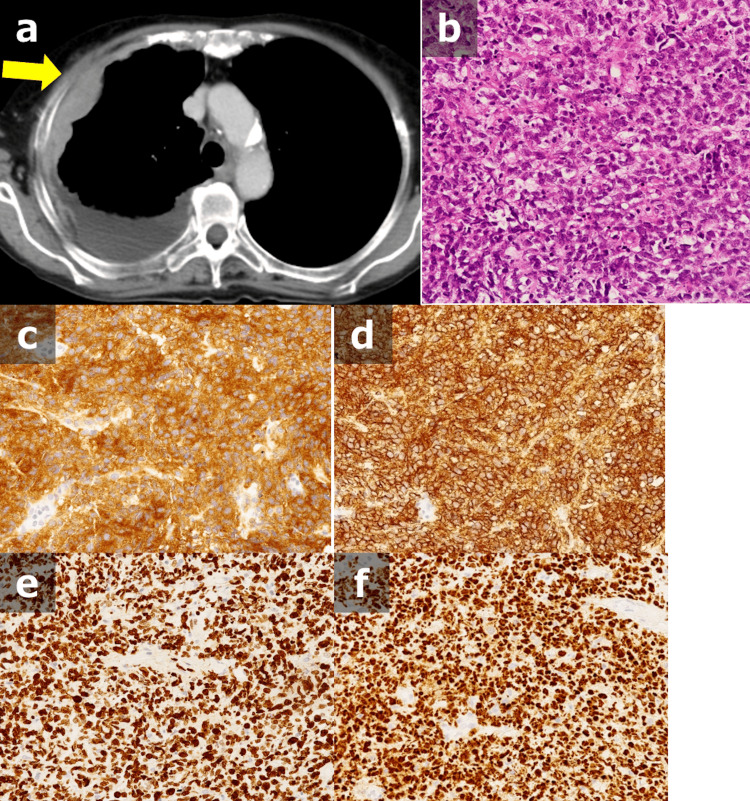
CT and pathological examination at onset (10 years prior) (a) CT revealed multiple swollen lymph nodes and right pleural masses (arrow). (b) A biopsy of the right pleural mass showed a dense proliferation of tumor cells with large, round nuclei. (c-f) Immunostaining showed positivity for CD10 (c), CD20 (d), and bcl-6 (e). Ki67 was 100% positive (f), but bcl-2 and EBER1 were negative. Burkitt's lymphoma was diagnosed CT: computed tomography

A 97-year-old woman was diagnosed with Burkitt’s lymphoma complicated by early-stage gastric cancer at the age of 87 years (10 years prior) in Sapporo City General Hospital. CT revealed multiple swollen lymph nodes in the neck, mediastinum, hilum, and abdomen, along with bilateral pleural effusions, a mass in the spleen, and right pleural masses. Several of the lymph nodes doubled in size within a month, measuring 27 mm in maximum diameter, and tended to be fused. A biopsy of the right pleural mass showed a dense proliferation of tumor cells with large, round nuclei. Immunostaining showed positive results for CD10, CD20, CD79a, and bcl-6, almost 100% positivity for Ki67, and negative results for pancytokeratin AE1/AE3, cyclin D1, bcl-2, and EBER1 (Figure 1).

Based on these results, Burkitt's lymphoma was diagnosed according to the fourth edition of the WHO classification [1]. The patient was treated with rituximab four times, and with pirarubicin, vincristine, cyclophosphamide, and prednisolone (THP-COP) once. The detailed treatment regimen for this case was as follows: 375 mg/m^2^ of rituximab on days 1, 8, 15, and 50 and THP-COP (50 mg/body pirarubicin, 1400 mg/body vincristine, and 750 mg/body cyclophosphamide) on days 14, representing approximately 75% of the standard dose. This dose was chosen due to the patient’s advanced age (87 years) and poor performance status (PS) of 3 on day 14. Nevertheless, chemotherapy was discontinued due to the adverse event of paralytic ileus. Neither the patient nor her family wanted further treatment for the lymphoma, and hence she received palliative care.

**Figure 2:**
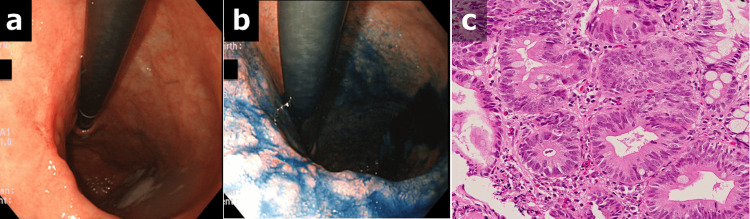
Esophagogastroduodenoscopy and pathological examination at onset (10 years prior) (a, b) A type 0-IIc lesion having an ulcer was seen in the middle of the greater curvature of the stomach, which was a contraindication for endoscopic resection. (c) Biopsy revealed well-differentiated adenocarcinoma

At that same time (at 87 years of age), esophagogastroduodenoscopy revealed a type 0-IIc lesion having an ulcer in the middle of the greater curvature of the stomach, and biopsy revealed well-differentiated adenocarcinoma (Figure 2).

As early-stage gastric cancer was not considered a prognostic factor compared to lymphoma, she initially received treatment for lymphoma. As chemotherapy was discontinued due to the adverse event and followed by pneumonia, mycoses, and pseudomembranous colitis, neither she nor her family wanted any treatments for gastric cancer. She was subsequently treated as an outpatient at Sapporo Shirakaba-dai Hospital. Four months after rituximab treatment, CT did not show any swollen lymph nodes or any masses in the spleen and right pleura.

Approximately 10 years after the initial treatment, at the age of 97, she was admitted to Sapporo Shirakaba-dai Hospital due to progressive deterioration in activities of daily living, accompanied by increasing malaise and dyspnea. Physical examination revealed tenderness in the right lower quadrant without signs of peritonitis. Laboratory data showed leukocytosis (10.0 x 10^9^/L), anemia (hemoglobin: 10.8 g/dL), high values of alkaline phosphatase, lactate dehydrogenase, and C-reactive protein (194 U/L, 387 U/L, and 5.65 mg/dL, respectively). Although both values of carcinoembryonic antigen and soluble interleukin-2 receptor (sIL-2R) were high at 22.5 ng/mL and 1600 U/mL, respectively, carbohydrate antigen 19-9 was normal at 8.4 U/mL (Table 1). 

**Table 1:** Laboratory data during final admission

Variable	Patient value	Unit	Reference range
Hemoglobin	10.8	g/dL	11.2–15.2
Total leukocyte count	10	x10^9^/L	3.5–9.7
Neutrophils	82.2	%	42.0-74.0
Monocytes	6.4	%	1.0-8.0
Lymphocytes	10.7	%	18.0-50.0
Platelet count	2.41	x10^12^/L	1.40-3.79
Erythrocyte sedimentation rate	71	mm/h	1-20
Albumin	3.2	g/dL	3.7-5.5
Total bilirubin	0.4	mg/dL	0.3-1.2
Aspartate aminotransferase	25	U/L	10-40
Lactate dehydrogenase	387	U/L	120-245
Alkaline phosphatase	194	U/L	38–113
Urea nitrogen	19.2	mg/dL	8.0-20.0
Creatinine	0.8	mg/dL	0.46-0.82
Uric acid	5.5	mg/dL	2.7-7.0
Sodium	138	mEq/L	135-145
Potassium	3.5	mEq/L	3.5-5.0
C-reactive protein	5.65	mg/dL	0-0.3
Carcinoembryonic antigen	22.5	ng/mL	0-5
Carbohydrate antigen 19-9	8.4	U/mL	0-37
Interleukin-2 receptor	1600	U/mL	122-496

**Figure 3:**
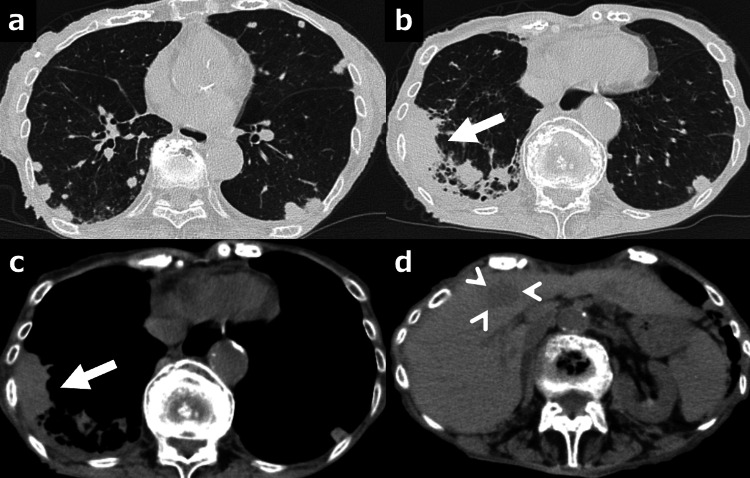
CT on final admission CT showed mass shadows in bilateral lung fields (a), a mass shadow (arrow) just below the pleura (b, c), and a mass shadow (arrowheads) in S4 of the liver (d) CT: computed tomography

**Figure 4:**
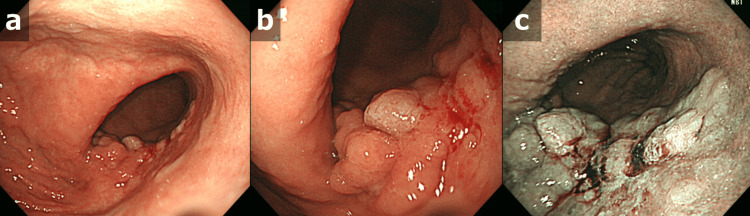
Esophagogastroduodenoscopy on final admission Esophagogastroduodenoscopy revealed a type 3 tumor in the midline of the gastric body: (a, b) white light, (c) narrowband image

CT at that time showed mass shadows in bilateral lung fields, a mass shadow just below the right lower pleura, and a mass shadow in S4 of the liver (Figure 3). Esophagogastroduodenoscopy revealed a type 3 tumor in the middle of the greater curvature of the stomach (Figure 4).

**Figure 5:**
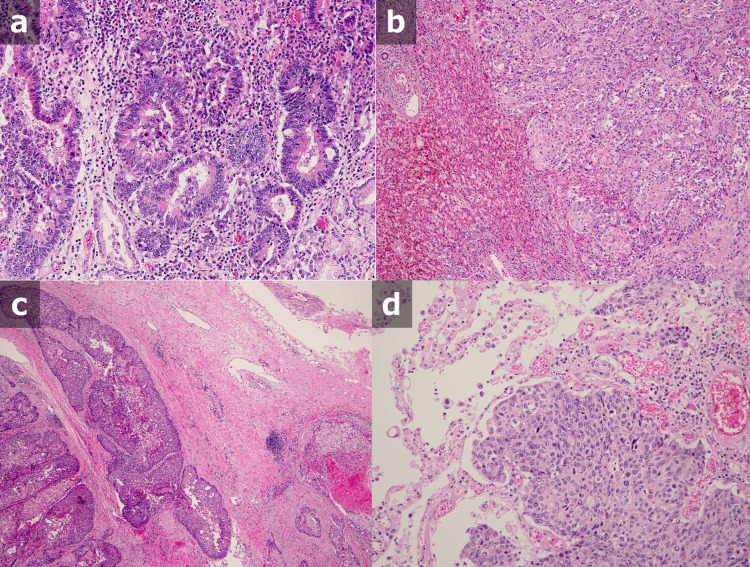
Pathological examination in autopsy The autopsy showed that the gastric cancer was adenocarcinoma (a), and all tumor cells, including in the liver (b), pleura (c), and lung (d) were also adenocarcinoma, not lymphoma cells

The patient received the best supportive care and subsequently died on hospital day 58. As the right pleural tumor was in a similar location to the previous lesion and sIL-2R was elevated, recurrence of lymphoma was suggested. An autopsy was performed with the consent of her family. The autopsy identified gastric cancer as adenocarcinoma, and all tumor cells identified from the lungs, pleura, and liver were also adenocarcinoma cells, not lymphoma cells (Figure 5).

**Figure 6:**
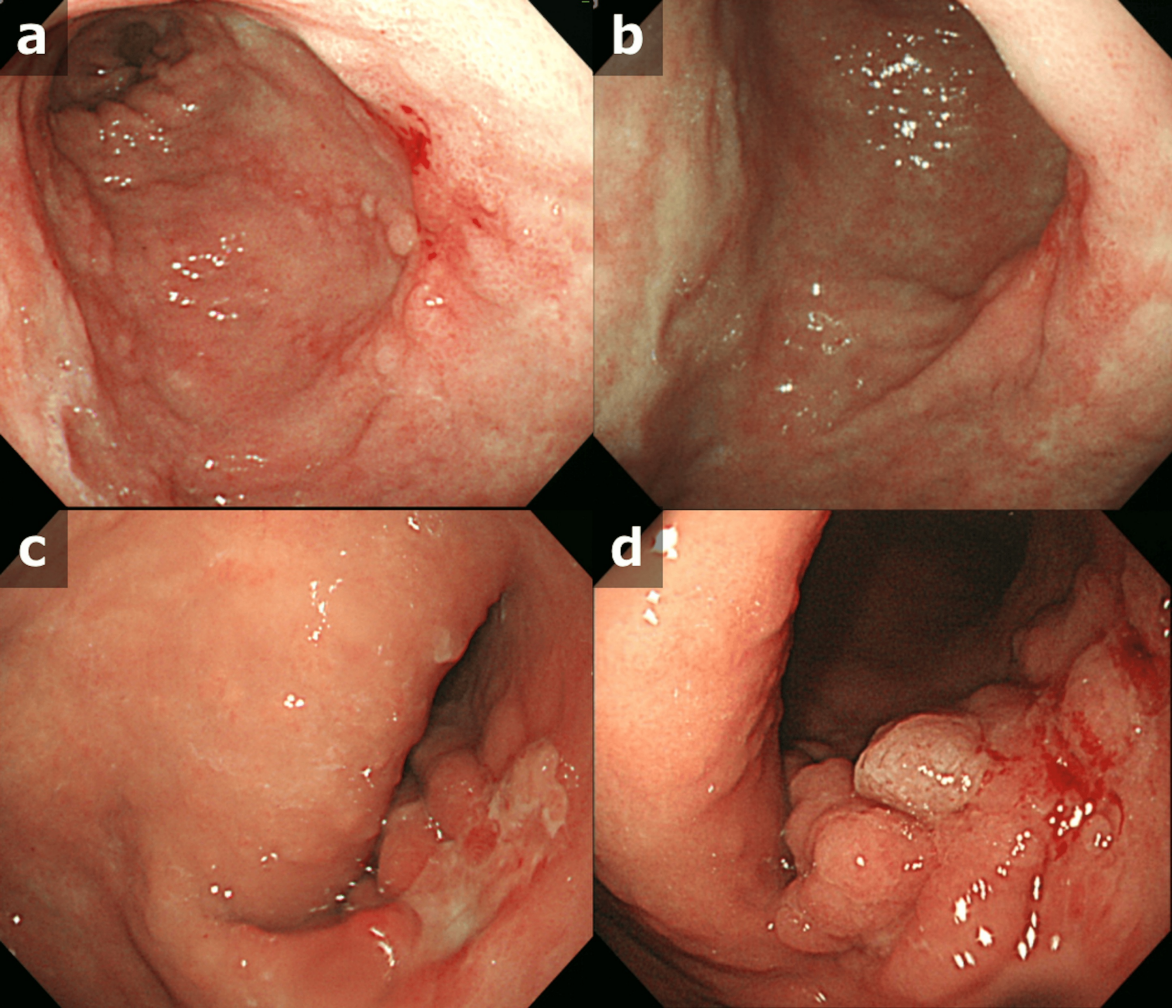
Clinical course of gastric cancer as per esophagogastroduodenoscopy A retrospective review of esophagogastroduodenoscopies revealed that the type 0-IIc lesion in the middle of the gastric body had gradually increased in size, the folds had become tighter, and the thickness had increased over 10 years. Even though we offered her treatments for gastric cancer several times, neither she nor her family wanted to undergo any. (a) 10 years earlier, (b) 7 years earlier, (c) 1 year earlier, and (f) on final admission

All tumors were thus considered to represent metastases of the gastric cancer, not recurrences of lymphoma. A retrospective review of the findings from esophagogastroduodenoscopy revealed that the type 0-IIc lesion in the middle of the gastric body had gradually increased in size, the folds had become tighter, and the thickness had increased over 10 years (Figure 6).

**Figure 7:**
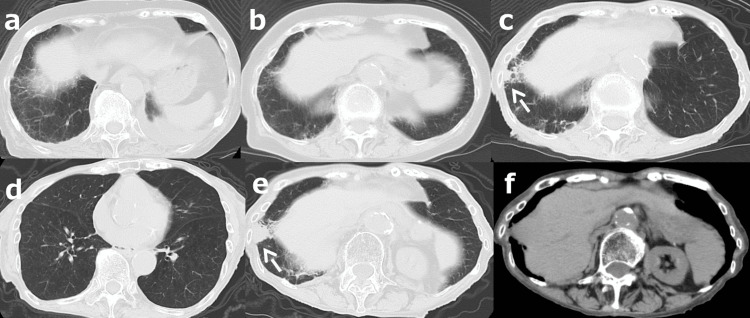
Clinical course as per CT A retrospective review of CT findings showed no mass in 10 (a) and 6 (b) years before the final admission and a tiny right pleural mass shadow (arrow) 1.5 years before the final admission (c), but no masses in either lung fields or the liver 8 months before final admission (d-f). The size of a right pleural mass (arrow) has become larger (e) CT: computed tomography

A retrospective review of CT findings showed a tiny right pleural mass 1.5 years before the final admission, but no masses in either lung or the liver eight months before final admission (Figure 7).

As the classification of lymphomas has been changed [1,2], we revised the diagnosis of lymphoma in this case. Immunohistochemistry showed that c-Myc was weakly positive in 30% of the cells and FISH did not show MYC translocation. Thus, the final diagnosis was changed to DLBCL, NOS.

## Discussion

We discuss a case of a patient with DLBCL who responded to rituximab-containing minimum chemotherapy. In this case, rituximab plus a single cycle of chemotherapy induced remission of DLBCL and the patient survived for 10 years without recurrence, suggesting rituximab as a potential treatment option for elderly individuals with DLBCL. In addition, this represents a valuable report as it illustrates an instance of early gastric cancer with 10 years of endoscopic follow-up.

The standard chemotherapy for CD20-positive advanced DLBCL involves six to eight cycles of the R-CHOP regimen (rituximab, cyclophosphamide, doxorubicin, vincristine, prednisone) [8]. However, it is difficult to conduct large-scale clinical trials in patients aged over 80 years, and optimal treatment strategies have not been established yet. The results of two meta-analyses suggest that reduced-dose R-CHOP therapy is preferable for elderly patients [9,10]. This patient was elderly and had a poor PS, and R-THP-COP, which was the standard treatment for Burkitt's lymphoma and DLBCL at the time, was administered.

There are two reports in the literature where rituximab monotherapy was effective for Burkitt's lymphoma. A 71-year-old Japanese man with Burkitt's lymphoma was successfully treated with rituximab monotherapy and remained in remission for more than three years without further lymphoma therapy [11]. Another report described a six-year-old boy with relapsed mediastinal Burkitt's lymphoma, with a tumor involving the bone marrow and both kidneys, who achieved a successful response to rituximab alone [12]. The efficacy of rituximab monotherapy for DLBCL has been reported, especially for elderly patients and those with severe/multiple comorbidities. Adjusted three-year overall survival of patients who received rituximab monotherapy was 48% and was higher than those without systemic therapy (18%) [13]. Patients who received rituximab monotherapy had a 69% decreased mortality risk compared to those without therapy [14].

There are also several case reports describing rituximab monotherapy successfully treating patients with DLBCL [15,16]. Although the hematological diagnosis changed from Burkitt’s lymphoma to DLBCL, NOS, this case remains one of lymphoma with high-grade malignancy that responded well to chemotherapy, including rituximab. In our case, rituximab 375 mg/m^2^ was administered four times (on days 1, 8, 15, and 50), and 75%-dose THP-COP was administered once (on day 14). Of course, the DLBCL may have responded to rituximab itself. However, the THP-COP might also have enhanced the effects of rituximab. In any case, rituximab appeared to exert a significant effect on tumor shrinkage. In addition, our patient was 87 years old with a poor PS of 3, suggesting a poor prognosis. Nevertheless, the fact that rituximab plus a single cycle of THP-COP reduced the tumor size and the patient remained alive and relapse-free for 10 years suggests its potential as a treatment option for the elderly.

The first study on the natural history of stomach cancer was published 70 years ago [17], and a few studies have since reported on the natural history of gastric carcinoma. Oh et al. reported the natural history of 101 patients with untreated gastric carcinoma, with five-year survival rates of 46.2% for stage I and 0% for stages II-IV [18]. Fujisaki et al. conducted a study involving an 8-year follow-up of patients with early gastric cancer complicated by severe heart disease, revealing that patients eventually died of heart failure [19]. Sato et al. reported a case of early gastric cancer that was followed for 12 years with the patient ultimately treated with submucosal dissection, and the cancer was identified as gastric cancer of the fundic gland type [20]. Our report involves a rare case without any specific treatment for gastric cancer in which endoscopic images were traceable over 10 years and the patient eventually died of tumor progression.

## Conclusions

Based on the fact that our patient survived for over 10 years, rituximab-containing minimum chemotherapy may be a useful treatment option for elderly patients with DLBCL with high-grade malignancy. In addition, this report constitutes a valuable contribution to the existing literature as it depicts a case where the progression of gastric cancer was observed endoscopically from the early to advanced stages.

## References

[1] Hasserjian RP, Ott G, Elenitoba-Johnson KS, Balague-Ponz O, de Jong D, de Leval L (2009). Commentary on the WHO Classification of Tumors of Lymphoid Tissues (2008): "Gray zone" lymphomas overlapping with Burkitt lymphoma or classical Hodgkin lymphoma. J Hematop.

[2] Alaggio R, Amador C, Anagnostopoulos I (2022). The 5th edition of the World Health Organization Classification of Haematolymphoid Tumours: Lymphoid Neoplasms. Leukemia.

[3] Lymphoma Study Group of Japanese Pathologists (2000). The World Health Organization Classification of Malignant Lymphomas in Japan: incidence of recently recognized entities. Pathol Int.

[4] Evens AM, Danilov A, Jagadeesh D (2021). Burkitt lymphoma in the modern era: real-world outcomes and prognostication across 30 US cancer centers. Blood.

[5] Nie M, Wang Y, Bi XW (2016). Effect of rituximab on adult Burkitt's lymphoma: a systematic review and meta-analysis. Ann Hematol.

[6] Crombie J, LaCasce A (2021). The treatment of Burkitt lymphoma in adults. Blood.

[7] Gordon MJ, Smith MR, Nastoupil LJ (2023). Follicular lymphoma: the long and winding road leading to your cure?. Blood Rev.

[8] Coiffier B, Lepage E, Briere J (2002). CHOP chemotherapy plus rituximab compared with CHOP alone in elderly patients with diffuse large-B-cell lymphoma. N Engl J Med.

[9] Beygi S, Sadashiv S, Reilly JB, Khan C, Lister J (2018). Frontline treatment of diffuse large B-cell lymphoma in elderly: a systematic review of clinical trials in post-rituximab era. Leuk Lymphoma.

[10] Bataillard EJ, Cheah CY, Maurer MJ, Khurana A, Eyre TA, El-Galaly TC (2021). Impact of R-CHOP dose intensity on survival outcomes in diffuse large B-cell lymphoma: a systematic review. Blood Adv.

[11] Nagasaki A, Yamanoha A, Okudaira T, Miyagi T, Takasu N (2009). Treatment-related Burkitt's lymphoma: literature review and case report of successful treatment with rituximab monotherapy. Acta Haematol.

[12] Okur FV, Oguz A, Karadeniz C, Citak C, Poyraz A, Boyunaga O (2006). Refractoriness to rituximab monotherapy in a child with relapsed/refractory Burkitt non-Hodgkin lymphoma. Pediatr Hematol Oncol.

[13] Tien YY, Link BK, Brooks JM, Wright K, Chrischilles E (2015). Treatment of diffuse large B-cell lymphoma in the elderly: regimens without anthracyclines are common and not futile. Leuk Lymphoma.

[14] Hamlin PA, Satram-Hoang S, Reyes C, Hoang KQ, Guduru SR, Skettino S (2014). Treatment patterns and comparative effectiveness in elderly diffuse large B-cell lymphoma patients: a surveillance, epidemiology, and end results-medicare analysis. Oncologist.

[15] Nakai S, Masaki T, Shintani T (2005). Diffuse large B-cell primary gastric lymphoma treated successfully with the CD20 monoclonal antibody (rituximab): a case with severe liver dysfunction due to liver cirrhosis and hepatocellular carcinoma. Oncol Rep.

[16] Pircher A, Gassner EM, Steurer M, Wolf D (2012). Durable complete remission in a patient with recurrent DLBCL receiving rituximab monotherapy after high-dose chemotherapy and autologous stem cell transplantation. BMJ Case Rep.

[17] Meiselas LE (1953). Observations on the natural history of gastric cancer. Am J Med Sci.

[18] Oh SY, Lee JH, Lee HJ (2019). Natural history of gastric cancer: observational study of gastric cancer patients not treated during follow-up. Ann Surg Oncol.

[19] Fujisaki J, Nakajima T, Hirasawa T (2012). Natural history of gastric cancer-a case followed up for eight years: early to advanced gastric cancer. Clin J Gastroenterol.

[20] Sato Y, Fujino T, Kasagawa A (2016). Twelve-year natural history of a gastric adenocarcinoma of fundic gland type. Clin J Gastroenterol.

